# In-air Hand Gesture Signature Recognition: An iHGS Database Acquisition Protocol

**DOI:** 10.12688/f1000research.74134.2

**Published:** 2023-05-02

**Authors:** Wee How Khoh, Ying Han Pang, Hui Yen Yap

**Affiliations:** 1 Faculty of Information Science and Technology, Multimedia University, Bukit Beruang, Melaka, 75450, Malaysia

**Keywords:** Dynamic Signature, Hand Gesture Signature, Gesture Recognition, Hand Gesture Signature Database, Image Processing, Forgeries Attack

## Abstract

**Background: **With the advances in current technology, hand gesture recognition has gained considerable attention. It has been extended to recognize more distinctive movements, such as a signature, in human-computer interaction (HCI) which enables the computer to identify a person in a non-contact acquisition environment. This application is known as in-air hand gesture signature recognition. To our knowledge, there are no publicly accessible databases and no detailed descriptions of the acquisitional protocol in this domain.

**Methods: **This paper aims to demonstrate the procedure for collecting the in-air hand gesture signature’s database. This database is disseminated as a reference database in the relevant field for evaluation purposes. The database is constructed from the signatures of 100 volunteer participants, who contributed their signatures in two different sessions. Each session provided 10 genuine samples enrolled using a Microsoft Kinect sensor camera to generate a genuine dataset. In addition, a forgery dataset was also collected by imitating the genuine samples. For evaluation, each sample was preprocessed with hand localization and predictive hand segmentation algorithms to extract the hand region. Then, several vector-based features were extracted.

**Results: **In this work, classification performance analysis and system robustness analysis were carried out. In the classification analysis, a multiclass Support Vector Machine (SVM) was employed to classify the samples and 97.43% accuracy was achieved; while the system robustness analysis demonstrated low error rates of 2.41% and 5.07% in random forgery and skilled forgery attacks, respectively.

**Conclusions: **These findings indicate that hand gesture signature is not only feasible for human classification, but its properties are also robust against forgery attacks.

## Introduction

A conventional dynamic signature recognition usually uses a special digitized device to capture the dynamic properties of a signature. A stylus pen is used to sign the signature on the surface of the digital tablet. This leaves a subtle track, exposing the signature information to others. A forger could learn the pattern from what they obtained from the tablet surface.

Numerous acquisition approaches have been proposed to replace the usage of a tablet for dynamic signatures. For instance, two ballpoint pens with sensors to measure the pen movement during the signing process, ^1^ a wearable device on the wrist (i.e. smartwatches) to capture the hand motion, ^2^ or an on-phone triaxial accelerometer built in a smartphone. ^3^^,^^4^

The introduction of low-cost sensor cameras ^5^ brings up new research opportunities for contactless human-computer interaction (HCI) in various applications such as robotics, healthcare, entertainment, intelligent surveillance, and intelligent environments. ^6^ Human hand gestures and dynamic signature recognition are becoming prevalent. This work proposes a hand gesture signature recognition system with the capability to recognize the identity of a person in a touchless acquisition environment. Additionally, a public database is provided for evaluation purposes.

Some relevant research works have been conducted using their own collected database. Tian et al. ^7^ introduced a Kinect-based password authentication system to explore the feasibility of a Kinect sensor to authenticate user-defined hand gesture passwords. In Ref. 8, the authors proposed a similar hand gesture signature recognition where the hand trajectory was used as the feature. The performance was evaluated on a self-collected database, consisting of 50 different classes. Empirical results demonstrated the feasibility and benefits of depth data in verifying a user’s identity based on a hand gesture signature. Fang et al. ^9^ proposed a fusion-based in-air signature verification. The user’s fingertip was tracked and the signature trajectory was extracted from a video sample captured by a high-speed camera. Malik et al. ^10^ implemented a neural network in recognizing hand gesture signatures for identity authentication. A CNN-based hand pose estimation algorithm was employed to estimate the hand joint position for the index fingertip. Multidimensional dynamic time warping (MD-DTW) was adopted to match the template and test signature data. It was tested on a self-collected dataset with 15 classes. The empirical results exhibited a promising recognition performance with the presence of depth features. Li and Sato ^11^ proposed an in-air signature authentication using the motion sensors of smart wrist-worn devices. The system captures signal-based gyroscope and accelerometer measurements employs a recurrent neural network (RNN) to classify between genuine and imposter hand signatures of twenty-two (22) participants. The research reported a highly promising equal error rate (EER) of only 0.83%. However, this research only tested the random forgeries of the signature.

From the literature, the existing studies were mainly utilizing their self-collected databases. To the best of our knowledge, there is no publicly available hand gesture signature database. The existence of a publicly available database can provide a freely available source of data to encourage more researchers into the field. For this reason, we present an openly available database, collected by the Microsoft Kinect sensor camera. To protect the privacy of the contributors, only depth information will be shared.

## Database collection

A Microsoft Kinect sensor camera is used as the main acquisition device to collect the samples of in-air hand gesture signature (iHGS) via its built-in IR projector and IR camera. A sample is a video clip that contains a set of image sequences disclosing the hand movement of a signature signing. The Kinect camera is capable of capturing up to 30 depth frames per second (fps). The number of image sequences (frames) of each sample corresponds to the duration of the hand movement and might be varying in each signature. Additionally, other computational factors such as heavy graphical processing and input latency affect the fps in each enrollment. These latencies may lead to a drop in the rate of fps, causing information loss. Thus, to ensure validation, the collected samples that have a fps rate of less than 27 are dropped/removed and the sample is re-captured through a similar procedure again. Figure 1 and Figure 2 depict the implementation of the iHGS sample acquisition process from both top and side views. The distances and spaces between the sensor camera and the subject were carefully chosen to ensure the entire body could be captured during the acquisition process. A more detailed data acquisition protocol can be found in Ref. 12.

**Figure 1.**
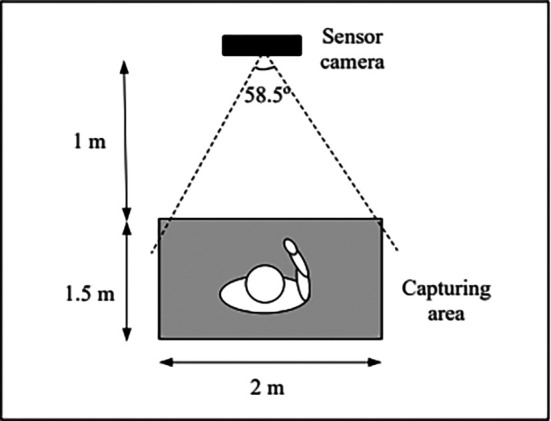
The top view of iHGS sample acquisition.

**Figure 2.**
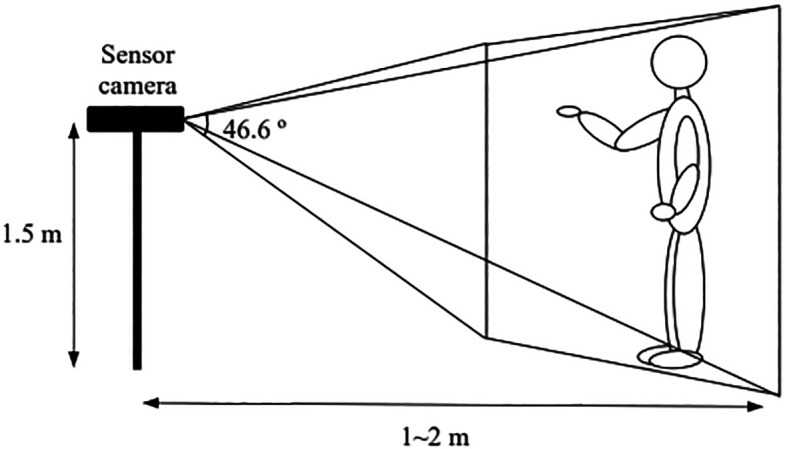
The side view of iHGS sample acquisition.

The database is named *iHGS database.* The data collection was conducted in two separate sessions and the entire process took four months to complete. Samples for the second session were collected with a time interval of approximately two to three weeks from the first session. This arrangement is intended to allow the intra-variances in genuine hand gesture signatures, better reflecting real-world situations. Before enrolment, the flow of the entire enrolment process was explained to each participant. They were given ample time to practice and familiarize themselves with the process before data acquisition.

A total of 100 participants were successfully enrolled. Among the participants, 69 were male and 31 female, aged from 18-40 years. 90% of participants were right-handed (signing with their right hand) with only 10% using their left hand (left-handed). Table 1 summarizes the characteristics of the iHGS database.

**Table 1.** Characteristics of hand gesture signature samples in the iHGS database.

Total number of participants			100
Male			69
Female			31
Age	18-19		12
	20-25		68
	26-30		8
	31-35		11
	36-40		1
Right-handed			90
Left-handed			10
Frame Rate			27-30 fps
No. of frame/signature	Genuine	Min	20
		Max	304
		Average	72.4
	Forgery	Min	21
		Max	294
		Average	76.6

There are two subsets of our *iHGS database*: (1) *genuine dataset*, and (2) *skilled forgery dataset.* For genuine dataset, each participant provides 10 genuine samples in each session (session 1 and session 2). A total of 2000 (10×2×100) samples were gathered for this genuine dataset.

A skilled forgery dataset contains forged signature samples. Each forger was provided with one genuine signature sample (signed by the genuine user on a piece of paper) randomly. They were asked to learn the signature with as much time as they needed. Then, each forger was asked to imitate the assigned signature 10 times. A total of 1000 skilled forgery signatures were successfully collected. However, 20 skilled forgery samples from two forgers (10 samples each) were corrupted due to the hardware error. Thus, only 980 skilled forgery samples were obtained. Table 2 summarizes the number of hand gesture signatures for the two subsets in the *iHGS database.*

**Table 2.** Summary of the number of hand gesture signatures for genuine and skilled forgery datasets.

Dataset		No. of samples
Genuine dataset	Total number of participants	100
	Number of samples/participants	20
	**Total samples**	**2000**
Skilled forgery dataset	Total number of forgers	98
	Number of forgery samples/forger	10
	**Total forgery samples**	**980**

## Methods

### Data preprocessing

Hand detection and localization techniques were applied to extract the region of interest (ROI) from each of the depth images of the iHGS database. A predictive hand segmentation technique was performed to precisely extract the hand region from the frames. Refer to Refs. 12, 13, 14 for more information.

### Feature generation

An iHGS sample is a collection of depth image sequences that comprises of *n* image frames, i.e. *n* is also the length of the sample. Several basic vector-based features are extracted from the sample. Firstly, a Motion History Image (MHI) process is performed on the preprocessed depth image sequence of each sample along the time. This technique effectively condenses the image sequence into a single grey-scale image (coined as *MHI* template), while preserving the motion information in a more compact form. ^15^^,^^16^ Specifically, *MHI* template describes the hand location and motion path along the time and generates a spatio-temporal information for the iHGS sample. The *MHI* image is then transformed into a vector space to produce a vector-based feature. The features explored in this work are as follows:

(a) *x-directional summation* ( ***V***_***X***_)

Produced by summing the *MHI* template in the vertical direction.

(b) *y-directional summation* ( ***V***_***Y***_)

Produced by summing the *MHI* template in the horizontal direction.

(c) *xy-directional summation* ( ***V***_***XY***_)

The concatenation of both *V _X_* and *V _Y_* features fora richer one-dimensional summation feature.

(d) Histogram of Oriented Gradient feature ( ***V***_***HOG***_)

A histogram descriptor is performed on the *MHI* template to extract the local texture, represented in a distribution of the edge and gradient structure. ^17^ It can discover the shape or the outline of the template image based on the slope or orientation gradient. It is worth noted that each pixel value in the *MHI* template describes the motion’s temporal information at a particular location. Thus, histogram orientation of the *MHI* template represents the intensity of motion history which is a useful feature.

(e) Binarized Statistical Image Features ( ***V***_***BSIF***_)

Statistical-based features are computed and summarized in a single histogram representation. First, the input image is convolved with a set of predefined filters to maximize the statistical independence of the filter responses. ^18^ Then, each response is applied to a nonlinear hashing operator to improve the computational efficiency. Next, the generated code map is regionalized into blocks and recapitulated into a block-wise histogram. These regional histograms are lastly concatenated into a global histogram, representing the underlying distribution of the data. In this work, different BSIF-based features are produced:

• ***V***_***BSIF-MHI***_ – *MHI* template is used as input data to the BSIF.
• ***V***_***BSIF-X***_ –Image sequences of an iHGS sample are projected along the *y*-axis to generate an *X-Profile* template. *X-Profile* template is used as input data to the BSIF.
• ***V***_***BSIF-Y***_ –Image sequences of an iHGS sample are projected along the *x*-axis to generate the *Y-Profile* template. *Y-Profile* template is used as input data to the BSIF.
• ***V***_***BSIF-XY***_ – Both *X-Profile* and *Y-Profile* templates are used as the data input to the BSIF.
• ***V***_***BSIF-MHIXY***_ – *MHI*, *X-Profile*, and *Y-Profile* templates are used as the data input to the BSIF.

## Experimental results

Two types of performance analyses are conducted: (1) *classification performance analysis*, and (2) *robustness analysis against forgery attacks.* A well-known multiclass Support Vector Machine (SVM) is adopted in the classification analysis through a One-versus-One (OVO) approach. The genuine dataset is randomly divided into a training set and a testing set with a ratio of *m:n* where *m* is larger than *n.* The training set is further partitioned into two subsets: validation subset and training subset with the ratio of *m _p_:n _q_.* The training subset is to train the SVM model; while the validation subset is to find the optimal model parameters for a minimal validation error. The model is then tested on the testing set for performance evaluation. The robustness performance analysis measures the security level against impersonation attempts. It demonstrates two attacks: random forgery and skilled forgery. In the former, a testing sample that belongs to a subject *i* is compared with all the remaining samples of other subjects in the genuine dataset. In the latter, a forged sample of a subject *j* (from the skilled forgery dataset) is matched with a claimed identity’s sample (i.e., genuine subject *i*’s sample) from the genuine dataset.

### Classification performance analysis

This analysis is implemented using the multi-class classification feature which is available in a library of SVM (LIBSVM) in MATLAB. ^19^ The samples of the genuine dataset are randomly partitioned into training, validation, and testing subsets, refer to Table 3.

**Table 3.** Data distribution in SVM classifier analysis.

**Genuine dataset**	Training samples	1000
	Validation samples	400
	Testing samples	600
	Total	2000

A polynomial kernel of the SVM classifier is utilized as part of our machine learning model. The samples were randomly partitioned into training, validation and testing subsets to evaluate the model’s performance. For cross-validation purposes, we repeated this random partitioning process five times using five different subsets. The hyperparameters for the polynomial kernel are tuned as such that the gamma (γ) is set to 20, the degree of the polynomial ( *d*) is set to 2 and the cost ( *C*) is set to 1. These hyperparameters were determined through empirical testing, and the settings that proposed yielded optimal and stable performance across our multiple experiments were used. The averaged classification measurements including *precision*, *recall*, *specificity*, and *F1-score* and the standard deviation are reported in Table 4. The accuracies among features are illustrated in Figure 1.

**Table 4.** Performances of precision, recall, specificity, and f1-score for polynomial kernel SVM.

Feature notation	Prec.	Recall	Spec.	F1-score
***V***_***x***_	63.82 ± 2.51	61.43 ± 2.00	99.61 ± 0.02	60.44 ± 2.19
***V***_***Y***_	64.94 ± 2.60	61.20 ± 2.15	99.61 ± 0.02	60.59 ± 2.19
***V***_***XY***_	88.45 ± 1.14	86.63 ± 1.16	99.87 ± 0.01	86.44 ± 1.32
***V***_***HOG***_	93.14 ± 0.70	91.63 ± 1.05	99.92 ± 0.01	91.63 ± 1.07
***V***_***BSIF-MHI***_	89.53 ± 1.32	88.03 ± 1.40	99.88 ± 0.01	87.83 ± 1.45
***V***_***BSIF-x***_	90.50 ± 1.48	88.87 ± 1.67	99.89 ± 0.02	88.74 ± 1.66
***V***_***BSIF-Y***_	92.20 ± 0.91	90.77 ± 0.77	99.91 ± 0.01	90.60 ± 0.83
***V***_***BSIF-XY***_	97.80 ± 0.30	97.43 ± 0.35	99.97 ± 0.00	97.42 ± 0.33
***V***_***BSIF-MHIXY***_	94.63 ± 0.56	93.57 ± 0.63	99.94 ± 0.01	93.55 ± 0.58

**Figure 1.**
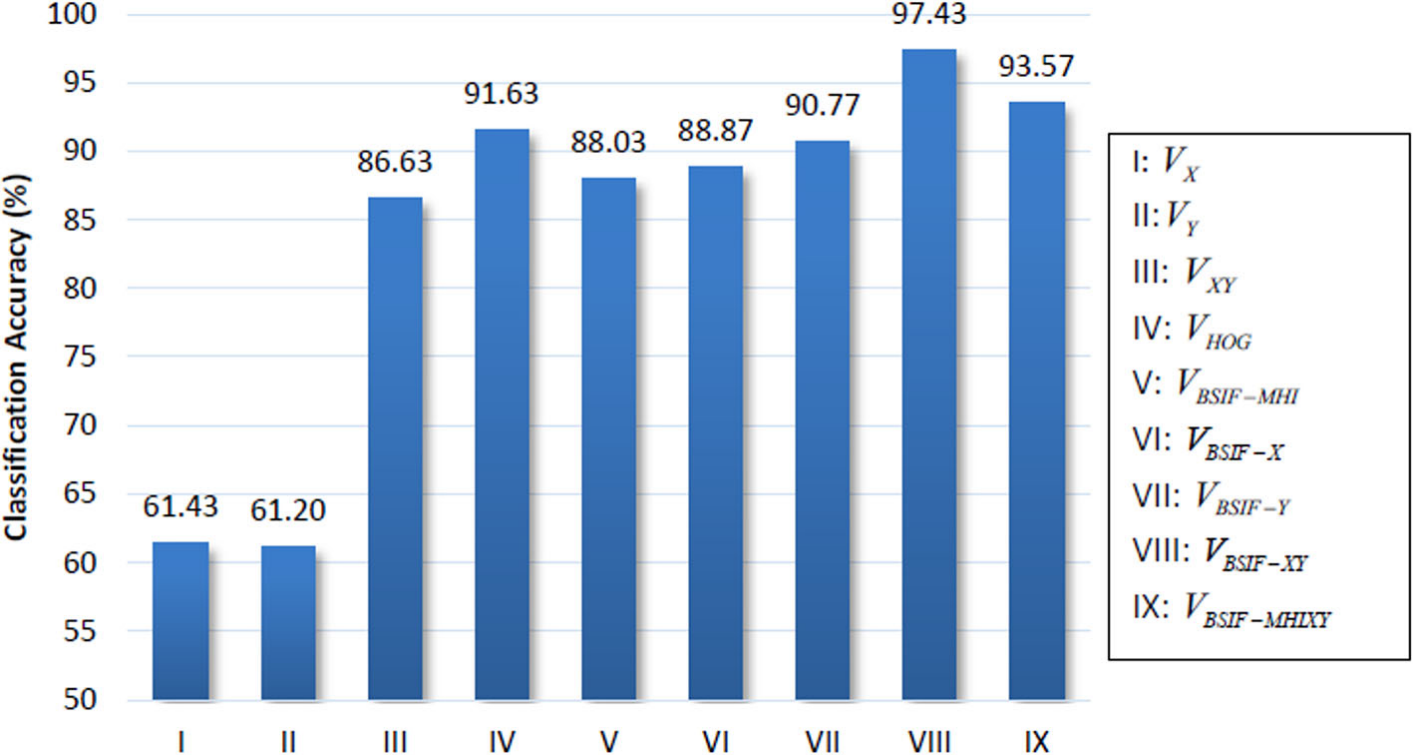
Classification accuracies of polynomial kernel SVM.

The classification results show the two BSIF features, *V _BSIF-XY_* and *V _BSIF-MHIXY_* achieving the best accuracy scores of 97.43% and 93.57%, respectively. It is followed by the HOG feature *V _HOG_* with an accuracy of 91.63%. It is noted that the system vaguely classifies the summation features, *V _X_* and *V _Y_* with accuracies of 61.43% and 61.20%. However, there is a boost in performance when concatenating them together, achieving 86.63% classification accuracy.

The results found that certain vector-based features such as *V _BSIF-XY_* and *V _BSIF-MHIXY_*, possess high levels of discriminative information for classifying in-air hand gesture signatures. Compared to other methods that involve complex preprocessing, the proposed vector-based features are extracted directly from the raw data, without the need for sophisticated techniques. These features can be used directly for classification model training, such as the SVM model, making it more convenient for real-world applications. Furthermore, the small value of standard deviation associated with these features suggests a high degree of stability in predicting hand gesture signatures. This is important in any classification task, as it ensures that the classification algorithm produces consistent and reliable results across a range of input data. The stability of these features is especially valuable in applications where the quality and consistency of the input data may vary. In summary, our findings demonstrate that vector-based features, particularly *V _BSIF-XY_* and *V _BSIF-MHIXY_*, offer a robust and reliable approach to iHGS classification. These features are easy to use and require minimal preprocessing, making them ideal for real-world applications that require efficient and accurate classification algorithms.

### Robustness performance analysis

This experimental analysis aimed to determine the robustness of the proposed approach against two types of forgery attacks, namely random forgery attacks and skilled forgery attacks.

The experiments were repeated for five trials. Averaged equal error rate (EER) and standard deviations were recorded. Four distance metrics were examined: Euclidean distance ( *EucD*), Cosine distance ( *CosD*), Chi-Square distance ( *CSqD*), and Manhattan distance ( *MD*).

Tables 5 and 6 report the system performances of two forgery attacks. It can be seen that the performances of the four kinds of distance metrics vary with different feature vectors. For the random forgery attack, *V _HOG_* with a cosine distance metric yields the lowest EER in random forgery (EER-R) of 2.41% followed by *V _BSIF-MHIXY_* with EER-R of 5.18%. Manhattan distance is not able to perform in this context as compared with the other metrics.

**Table 5.** EER for random forgery attack (EER-R).

Feature notation	EER-R (AVG% ± STD)			
	*EucD*	*CosD*	*CSqD*	*MD*
***V***_***x***_	14.33 ± 0.16	8.64 ± 0.35	10.36 ± 0.21	15.01 ± 0.41
***V***_***Y***_	11.87 ± 0.30	7.33 ± 0.30	8.54 ± 0.33	13.08 ± 0.22
***V***_***XY***_	10.58 ± 0.22	2.91 ± 5.07	6.62 ± 0.11	11.55 ± 0.32
***V***_***HOG***_	21.96 ± 0.34	**2.41 ± 0.22**	19.49 ± 0.66	25.74 ± 0.24
***V***_***BSIF-MHI***_	5.94 ± 0.37	6.35 ± 0.28	9.88 ± 0.21	13.00 ± 0.30
***V***_***BSIF-x***_	10.51 ± 0.37	9.44 ± 0.69	7.99 ± 0.49	12.09 ± 0.44
***V***_***BSIF-Y***_	10.10 ± 0.57	10.33 ± 0.24	9.92 ± 0.41	14.02 ± 0.19
***V***_***BSIF-XY***_	8.84 ± 0.43	7.01 ± 0.39	7.86 ± 0.56	12.19 ± 0.28
***V***_***BSIF-MHIXY***_	5.18 ± 0.28	5.43 ± 0.10	7.49 ± 0.26	11.15 ± 0.32

**Table 6.** EER for skilled forgery attack (EER-S).

Feature notation	EER-S (AVG% ± STD)			
	*EucD*	*CosD*	*CSqD*	*MD*
***V***_***x***_	18.97 ± 0.25	15.00 ± 0.85	15.39 ± 0.42	19.44 ± 0.53
***V***_***Y***_	14.70 ± 0.37	10.31 ± 0.32	11.11 ± 0.23	15.47 ± 0.37
***V***_***XY***_	15.01 ± 0.32	**5.07 ± 0.23**	10.25 ± 0.43	16.87 ± 0.22
***V***_***HOG***_	25.69 ± 0.32	**5.07 ± 0.53**	24.42 ± 0.58	29.71 ± 0.42
***V***_***BSIF-MHI***_	9.45 ± 0.64	10.43 ± 0.43	15.40 ± 0.60	19.62 ± 0.46
***V***_***BSIF-x***_	20.59 ± 0.50	18.64 ± 0.87	19.39 ± 0.63	23.62 ± 0.81
***V***_***BSIF-Y***_	16.52 ± 0.60	16.55 ± 0.73	16.39 ± 0.43	20.87 ± 0.28
***V***_***BSIF-XY***_	16.16 ± 0.67	13.99 ± 0.61	15.42 ± 0.41	21.59 ± 0.57
***V***_***BSIF-MHIXY***_	9.47 ± 0.67	9.84 ± 0.23	14.97 ± 0.20	19.00 ± 0.50

Distinguishing skilled forgery attacks from genuine signatures is undeniably more challenging than detecting random forgery attacks, due to the high similarity between the forgery and genuine samples. Consequently, the Equal Error Rates (EERs) for skilled forgery attacks are expected to be higher than for random forgery attacks. Our study found that the vector-based features *V _XY_* and *V _HOG_*, when adopted with the cosine distance metric, achieved the best EER-S of 5.07% for skilled forgery attacks. This is a promising result, and it proves that these features can be effective in distinguishing skilled forgeries from genuine signatures. *V _BSIF-MHIXY_* with the Euclidean distance metric, obtained an EER-S of 9.45%, which is also a relatively good result. On the other hand, most BSIF features were found to perform poorly in verifying skilled forged hand gesture signatures, highlighting the importance of carefully selecting the features used for authentication. Similar to random forgery attacks, the Manhattan distance metric achieved the worst performance. Again, it indicates that the selection of the right distance metric is crucial for achieving good verification performance. In summary, these findings demonstrate that the verification performance of iHGS is not solely determined by the extracted features but is also highly dependent on the choice of distance metric. Therefore, careful consideration must be given to both factors in verifying the iHGS.

## Conclusions

In this paper, we presented a self-collected *iHGS database* and a detailed description of the acquisition protocol to collect the database. Several basic sets of vector-based features were extracted from the samples. This paper also investigated the effectiveness of classification capability as well as the robustness against forgery attacks. The experimental results for both analyses have shown promising results with the appropriate features extracted from the samples. Our analyses demonstrate the potential of iHGS in both recognition and verification. However, there is room for future exploration in iHGS. The current database was collected in a controlled environment. As a biometric authentication, other external factors such as angles of the camera, the distance between user and acquisition devices, different background complexity, etc should be considered. In particular, it could be further extended by considering those uncontrolled environmental factors to increase the challenge of the database.

## Data availability and materials

Figshare: In-air Hand Gesture Signature Database (iHGS Database) https://doi.org/10.6084/m9.figshare.16643314

This project contains the following underlying data:

• Genuine dataset (100 contributors labels with ID from 1 to 100)
• Skilled forgery dataset (98 contributors labels with ID from 1 to 100 where ID of 84 and 88 are not included)

Data are available under the terms of the Creative Commons Attribution 4.0 International license (CC-BY 4.0).

## Ethics approval and consent to participate

The experimental analyses were established, according to the ethical guideline and were approved by the Research Ethics Committee (REC) with the ethical approval number EA1452021. Written informed consent was obtained from individual participants.

## Author contributions

W.H. carried out the experiment with support from Y.H. and H.Y. coordinated the data collection and establishment of the database. Besides, W.H. took the lead in writing the manuscript while Y.H. and H.Y. provided critical feedback and helped shape the analysis and manuscript.
