# Supplementary material for: In-air Hand Gesture Signature Recognition: An iHGS Database Acquisition Protocol
Source: F1000Res. 2023 May 2;11:283. Originally published 2022 Mar 7. [Version 2] doi: 10.12688/f1000research.74134.2 (PMC10439358; doi:10.12688/f1000research.74134.2)
Supplement: Peer review history

# Peer review: Reviewer response for version 2

**DOI:** 10.5256/f1000research.147166.r189966

**Published:** 2023-08-18

**Authors:** Paolo Roselli, Jean Vanderdonckt

**Affiliations:** 1 Università di Roma, Rome, Italy; 2 Louvain School of Management, Universite catholique de Louvain, Louvain-la-Neuve, Walloon Region, Belgium

The short article briefly describes a particular way to collect in-air hand gesture signatures to produce a publicly accessible database of such in-air hand gesture signatures.

 - Are sufficient details of methods and analysis provided to allow replication by others? I answered "partly" because: 

1. It is not clear what "skilled" precisely means 
2. It is not clear if the set of participants producing the "Genuine" dataset is disjoint from the set of forgers producing the "Skilled forgery" dataset
3. A visual example of in-air signature would have been appreciated

- If applicable, is the statistical analysis and its interpretation appropriate?

- I answered "partly" because in "Figure 1" I would have appreciated to see the confidence intervals 

- Are the conclusions drawn adequately supported by the results?

- I answered "partly" because the term "human classification" is open to interpretations and applications too varied and potentially dangerous to humans to be simply considered as "feasible". The same consideration can be made about the claim "robust against forgery attacks".

Is the work clearly and accurately presented and does it cite the current literature?

Yes

If applicable, is the statistical analysis and its interpretation appropriate?

Partly

Are all the source data underlying the results available to ensure full reproducibility?

Yes

Is the study design appropriate and is the work technically sound?

Yes

Are the conclusions drawn adequately supported by the results?

Partly

Are sufficient details of methods and analysis provided to allow replication by others?

Partly

Reviewer Expertise:

Vector based gesture recognition; Mathematical Analysis; Clifford Geometric Algebra

We confirm that we have read this submission and believe that we have an appropriate level of expertise to confirm that it is of an acceptable scientific standard.

# Peer review: Reviewer response for version 1

**DOI:** 10.5256/f1000research.77854.r126552

**Published:** 2022-12-12

**Authors:** Cheng-Yaw Low

**Affiliations:** 1 Institute for Basic Science, Daejeon, South Korea

This manuscript contributes a small-scale in-air hand gesture signature dataset to the research community.

 However, this manuscript is overly simplified. 

 My concerns are as follows:

1. The authors should compare the new dataset to other repositories, e.g. the ones employed in Refs. 11, 12, etc. Despite of being private, these existing datasets should be at least introduced in the manuscript. Moreover, the main reason why this newly constructed dataset is important to iHGS should further be underlined. 
2. I think some relevant illustrations should be provided?
3. Some parts in the Method section are not clear, e.g., the data preprocessing section, the feature dimensions for V_HoG and V_BSIF are not disclosed, etc.
4. The following statement is problematic?
*"The data distribution is randomized in five different trials using a polynomial kernel."*

Therefore, a revision is needed prior to indexing.

Is the work clearly and accurately presented and does it cite the current literature?

Yes

If applicable, is the statistical analysis and its interpretation appropriate?

Partly

Are all the source data underlying the results available to ensure full reproducibility?

Partly

Is the study design appropriate and is the work technically sound?

Partly

Are the conclusions drawn adequately supported by the results?

Partly

Are sufficient details of methods and analysis provided to allow replication by others?

Partly

Reviewer Expertise:

Biometrics, Pattern Recognition.

I confirm that I have read this submission and believe that I have an appropriate level of expertise to confirm that it is of an acceptable scientific standard, however I have significant reservations, as outlined above.

# Response

**Published:** 2023-04-26

**Authors:** Wee How Khoh

**Affiliations:** Multimedia University, Malaysia

We would like to express our sincerest gratitude to you and to all the reviewers for their constructive comments on our manuscript entitled “In-air Hand Gesture Signature Recognition: An iHGS Database Acquisition Protocol” and thanks for the opportunity to revise the paper. We have incorporated changes that reflect the detailed suggestions you have graciously provided. We also hope that our edits and the responses below satisfactorily address all the issues and concerns you and the reviewers have noted.

**1.** *"The authors should compare the new dataset to other repositories, e.g. the ones employed in Refs. 11, 12, etc. Despite of being private, these existing datasets should be at least introduced in the manuscript. Moreover, the main reason why this newly constructed dataset is important to iHGS should further be underlined."*

**Response:**

 Thank you for your valuable feedback. We appreciate your comment in our manuscript and would like to clarify that the datasets mentioned in Refs. 11 and 12 are identical to ours in this manuscript. It's important to note that, to date, there are no publicly available in-air hand gesture signature datasets, which makes it challenging to compare our dataset to them. Our manuscript aims to address this gap in the literature by presenting the acquisition protocol used to build the dataset and share the dataset with the public. We hope that this will contribute to further research in this area. Thank you once again for your feedback.

**2.** *"I think some relevant illustrations should be provided?"*

**Response:**

 Thank you for your comment. We have updated the manuscript accordingly. Figure 1 and Figure 2 have been included in the revised version to illustrate the iHGS sample acquisition process from both top and side views. We hope these figures will help clarify the methodology and improve the manuscript's overall readability.

**3.** *"Some parts in the Method section are not clear, e.g., the data preprocessing section, the feature dimensions for V_HoG and V_BSIF are not disclosed, etc."*

**Response:**

 We agree that providing more details can enhance the understanding of our research. However, due to the page constraint of the current manuscript, we were unable to include all the necessary information and details. To address this, we have added a statement to the manuscript (in the section “Method”), directing readers to our previous research paper Ref. 12, 13 and 14, where a more comprehensive description of the methodology can be found.

**4.** " *The following statement is problematic?*

*"The data distribution is randomized in five different trials using a polynomial kernel."*"

**Response:**

 Thank you for your comment. We apologize for any confusion and misleading in the statement. We have revised the statement as follows:

**Revised texts:**

 A polynomial kernel of the SVM classifier is utilized as part of our machine learning model. The samples were randomly partitioned into training, validation and testing subsets to evaluate the model’s performance. For cross-validation purposes, we repeated this random partitioning process five times using five different subsets.

# Peer review: Reviewer response for version 1

**DOI:** 10.5256/f1000research.77854.r126550

**Published:** 2022-03-30

**Authors:** Lillian Yee Kiaw Wang

**Affiliations:** 1 Monash University Malaysia, Subang Jaya, Malaysia

The article discussed how a self-collected iHGS database is built. The acquisition protocol is clearly described. The effectiveness of classification capability and robustness against forgery attacks are also discussed. 

 Some points for your consideration:

1. Some of the threshold values stated in the article should be supported by citation or justification on why that particular value is selected. For example:
  - "Thus, to ensure validation, the collected samples that have an fps rate less than 27 are removed and re-captured again" Needs citation or justification, why 27?
  - "The optimal hyperparameters for the polynomial kernel are tuned empirically such that the gamma (γ) is set to 20, the degree of the polynomial ( *d*) is set to 2 and the cost ( *C*) is set to 1." Needs citation or justification, why the parameters were set as such?
2. The discussion of results for classification performance analysis is quite brief. Suggest to elaborate further on its impacts, for example, easy learning process and prediction stability -- easy or stable in what way? Also, apparent significance of your results compared to previous research works can be discussed in detail.
3. Similar comments for the discussion of results for robustness performance analysis (refer point 2).
4. More update-to-date references would be good, particularly references to support the discussions of result should be of recent research works.

Is the work clearly and accurately presented and does it cite the current literature?

Partly

If applicable, is the statistical analysis and its interpretation appropriate?

Not applicable

Are all the source data underlying the results available to ensure full reproducibility?

Yes

Is the study design appropriate and is the work technically sound?

Yes

Are the conclusions drawn adequately supported by the results?

Yes

Are sufficient details of methods and analysis provided to allow replication by others?

Yes

Reviewer Expertise:

IoT, Education, Technology Acceptance, Data Analytics

I confirm that I have read this submission and believe that I have an appropriate level of expertise to confirm that it is of an acceptable scientific standard.

# Response

**Published:** 2023-04-26

**Authors:** Wee How Khoh

**Affiliations:** Multimedia University, Malaysia

We would like to express our sincerest gratitude to you and to all the reviewers for their constructive comments on our manuscript entitled “In-air Hand Gesture Signature Recognition: An iHGS Database Acquisition Protocol” and thanks for the opportunity to revise the paper. We have incorporated changes that reflect the detailed suggestions you have graciously provided. We also hope that our edits and the responses below satisfactorily address all the issues and concerns you and the reviewers have noted.

**1.** *"Some of the threshold values stated in the article should be supported by citation or justification on why that particular value is selected. For example:*

- *"Thus, to ensure validation, the collected samples that have an fps rate less than 27 are removed and re-captured again" Needs citation or justification, why 27?*
- *"The optimal hyperparameters for the polynomial kernel are tuned empirically such that the gamma (γ) is set to 20, the degree of the polynomial (d) is set to 2 and the cost (C) is set to 1." Needs citation or justification, why the parameters were set as such?"*

**Response:**

 Thank you for the comment.

 We removed signature samples with a frame-per-second (fps) rate of less than 27 out of 30 in order to maintain the quality of the collected database. This decision was made after careful consideration to ensure that the database is valid and not affected by hardware constraints.

 For the second point, the hyperparameters of the kernel were determined through empirical testing, and the settings we used were found which yielded optimal and stable performances across multiple experiments. Justification has been added to the manuscript.

**Revised texts:**

 The hyperparameters for the polynomial kernel are tuned as such that the gamma (γ) is set to 20, the degree of the polynomial (d) is set to 2 and the cost (C) is set to 1. These hyperparameters were determined through empirical testing, and the settings that proposed yielded optimal and stable performance across our multiple experiments were used.

**2.** *"The discussion of results for classification performance analysis is quite brief. Suggest to elaborate further on its impacts, for example, easy learning process and prediction stability -- easy or stable in what way? Also, apparent significance of your results compared to previous research works can be discussed in detail."*

**Response:**

 To resolve the confusion of the use of the “easy learning process”, we have rephrased the paragraph. This paper mainly focuses on the acquisition protocol adapted to record and collect the iHGS samples. A detailed procedure and steps of the acquisition protocol have been outlined. After thorough consideration, we decided not to include the comparison of previous research works as it has already been included in our previous research paper.

**Revised texts:**

 The results found that certain vector-based features such as VBSIF-XY and VBSIF-MHIXY, possess high levels of discriminative information for classifying in-air hand gesture signatures. Compared to other methods that involve complex preprocessing, the proposed vector-based features are extracted directly from the raw data, without the need for sophisticated techniques. These features can be used directly for classification model training, such as the SVM model, making it more convenient for real-world applications.Furthermore, the small value of standard deviation associated with these features suggests a high degree of stability in predicting hand gesture signatures. This is important in any classification task, as it ensures that the classification algorithm produces consistent and reliable results across a range of input data. The stability of these features is especially valuable in applications where the quality and consistency of the input data may vary. In summary, our findings demonstrate that vector-based features, particularly VBSIF-XY and VBSIF-MHIXY, offer a robust and reliable approach to iHGS classification. These features are easy to use and require minimal preprocessing, making them ideal for real-world applications that require efficient and accurate classification algorithms.

**3. *"*** *Similar comments for the discussion of results for robustness performance analysis (refer point 2)."*

**Response:**

 Thank you for your feedback. We have revised the discussion of results for robustness performance analysis.

**Revised texts:**

 Distinguishing skilled forgery attacks from genuine signatures is undeniably more challenging than detecting random forgery attacks, due to the high similarity between the forgery and genuine samples. Consequently, the Equal Error Rates (EERs) for skilled forgery attacks are expected to be higher than for random forgery attacks. Our study found that the vector-based features V XY and V HOG, when adopted with the cosine distance metric, achieved the best EER-S of 5.07% for skilled forgery attacks. This is a promising result, and it proves that these features can be effective in distinguishing skilled forgeries from genuine signatures.  V BSIF-MHIXY with the Euclidean distance metric, obtained an EER-S of 9.45%, which is also a relatively good result. On the other hand, most BSIF features were found to perform poorly in verifying skilled forged hand gesture signatures, highlighting the importance of carefully selecting the features used for authentication. Similar to random forgery attacks, the Manhattan distance metric achieved the worst performance. Again, it indicates that the selection of the right distance metric is crucial for achieving good verification performance. In summary, these findings demonstrate that the verification performance of iHGS is not solely determined by the extracted features but is also highly dependent on the choice of distance metric. Therefore, careful consideration must be given to both factors in verifying the iHGS.

**4.** *"More update-to-date references would be good, particularly references to support the discussions of result should be of recent research works."*

**Response:**

 Thank you for your comment. This paper focuses on the acquisition protocol of iHGS, which has not been widely researched in recent years, especially regarding the methodology of collecting and establishing the benchmark dataset. Therefore, we have limited our review to similar works related to hand gesture signatures and have incorporated the latest related research into the paper.
